# Geographical variation in ADHD: do diagnoses reflect symptom levels?

**DOI:** 10.1007/s00787-022-01996-7

**Published:** 2022-05-18

**Authors:** Tarjei Widding-Havneraas, Simen Markussen, Felix Elwert, Ingvild Lyhmann, Ingvar Bjelland, Anne Halmøy, Ashmita Chaulagain, Eivind Ystrom, Arnstein Mykletun, Henrik Daae Zachrisson

**Affiliations:** 1 Department of Clinical Medicine, University of Bergen, Jonas Lies Vei 87, 5021 Bergen, Norway; 2 Centre for Research and Education in Forensic Psychiatry, Haukeland University Hospital, Bergen, Norway; 3 Ragnar Frisch Centre for Economic Research, Oslo, Norway; 4 Department of Sociology, University of Wisconsin-Madison, Madison, WI USA; 5 Division of Psychiatry, Haukeland University Hospital, Bergen, Norway; 6 Promenta Research Center, Department of Psychology, University of Oslo, Oslo, Norway; 7 Department of Mental Disorders, Norwegian Institute of Public Health, Oslo, Norway; 8 Division of Health Services, Norwegian Institute of Public Health, Oslo, Norway; 9 Department of Community Medicine, University of Tromsø, Tromsø, Norway; 10 Centre for Work and Mental Health, Nordland Hospital, Bodø, Norway; 11 Department of Special Needs Education, University of Oslo, Oslo, Norway

**Keywords:** Health services, Psychiatry, Child health, Adolescent, Norwegian mother, father and child cohort study, MoBa, Norwegian patient registry, ADHD, Symptoms

## Abstract

Rates of ADHD diagnosis vary across regions in many countries. However, no prior study has investigated how much within-country geographic variation in ADHD diagnoses is explained by variation in ADHD symptom levels. We examine whether ADHD symptom levels explain variation in ADHD diagnoses among children and adolescents using nationwide survey and register data in Norway. Geographical variation in incidence of ADHD diagnosis was measured using Norwegian registry data from the child and adolescent mental health services for 2011–2016. Geographical variation in ADHD symptom levels in clinics’ catchment areas was measured using data from the Norwegian mother, father and child cohort study for 2011–2016 (*n* = 39,850). Cross-sectional associations between ADHD symptom levels and the incidence of ADHD diagnoses were assessed with fractional response models. Geographical variation in ADHD diagnosis rates is much larger than what can be explained by geographical variation in ADHD symptoms levels. Treatment in the Norwegian child and adolescent mental health services is free, universally available upon referral, and practically without competition from the private sector. Factors beyond health care access and unequal symptom levels seem responsible for the geographical variation in ADHD diagnosis.

**Supplementary Information:**

The online version contains supplementary material available at 10.1007/s00787-022-01996-7.

## Introduction

**Fig. 1:**
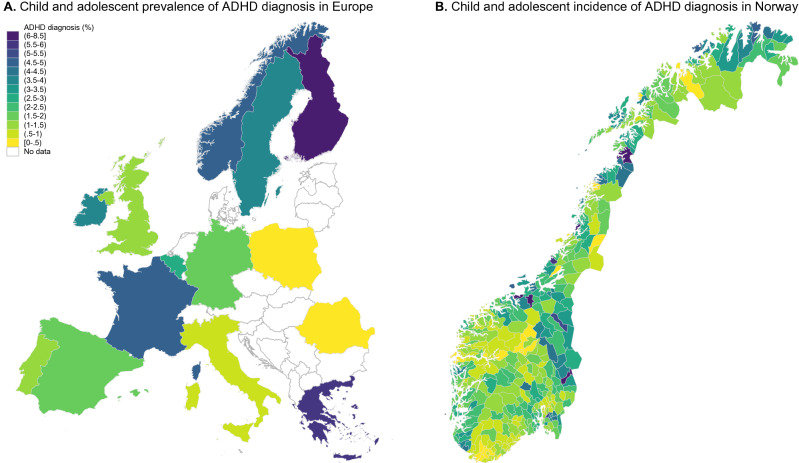
Geographical variation in ADHD diagnosis in Europe and Norway. Panel **A** Prevalence rate of ADHD diagnosis in children and adolescents across European countries. Panel **B** Incidence rate of ADHD diagnosis in Norway, 0–18 years, 2011–2016. ADHD diagnoses registered in the Norwegian Patient Registry by municipality (*n* = 428)

Diagnosis rates of attention-deficit/hyperactivity disorder (ADHD) vary across many countries [1]. International comparisons suffer validity problems due to differing diagnostic standards and methodology (Fig. 1A) [1]. Similar geographic variation in diagnostic prevalence, however, exists within countries with a uniform diagnostic standard, for example, Norway (Fig. 1B) [2–6].

Although ADHD symptoms are fundamental in diagnosing ADHD, no prior research investigates the extent to which geographical variation in ADHD symptoms explain geographical variation in ADHD diagnoses. ADHD diagnosis is a precondition for ADHD treatment, especially pharmacological treatment. Geographical variation in ADHD diagnoses that does not correlate with variation in symptoms would thus raise concerns about over- and undertreatment of ADHD [7–10].

We study the extent to which geographic variation in ADHD symptoms explains geographic variation in ADHD diagnosis in Norway, where 5% of children and adolescents are diagnosed with ADHD [11]. Studying Norway has three distinct advantages. First, the availability of comprehensive, nationwide, and geo-coded data on ADHD symptoms and diagnosis. Specifically, we combine nationwide survey data on ADHD symptoms from the Norwegian mother, father and child cohort study (MoBa) with nationwide register data on the incidence of ADHD diagnosis from the Norwegian Patient Registry (NPR). Second, Norway’s universal and free health care system (with a marginal private sector) largely rules out variation in healthcare access as an explanation for variation in ADHD diagnosis. Third, nationwide diagnostic standards largely rule out another explanation for geographical variation in ADHD diagnosis. Norwegian child and adolescent mental health services (CAMHS) are organized by clinics serving catchment areas comprised of one or more municipalities/city districts, where only specialists diagnose patients with ADHD and initiate treatment using national treatment guidelines [12].

The aim of this study is to examine whether ADHD symptom levels explain variation in ADHD diagnoses among children and adolescents. We explore three research questions: (1) Does between-clinics variation in the incidence rate of ADHD diagnosis exceed chance variation? (2) Does between-clinics variation in symptom levels of ADHD exceed chance variation? (3) Does between-clinics variation in the incidence rate of ADHD diagnosis, conditional on symptoms levels of ADHD, exceed chance variation?

## Methods

### ADHD symptoms

We measured ADHD symptoms levels for the general population using mother-reported data from MoBa for 2011–2016. We used two measures of ADHD symptoms: (1) The proportion of the population in a clinics’ catchment area with ADHD symptoms equals to or above the 95th percentile, in line with a country prevalence of 5% among children and adolescents [11]. (2) As a sensitivity measure, we used a 90th percentile cut-off as some clinics may be more prone to diagnose ADHD than others. Proportions of children with high ADHD symptom levels were calculated with individuals who scored above the thresholds as the numerator and the total participants as the denominator.

Data were reported when the child was 8 years old, corresponding to the average age of diagnosis in Norway [13]. The Parent/Teacher Rating Scale for Disruptive Behavior Disorders (RS-DBD) was used, which has good instrument validity and reliability [14, 15], and corresponds with rating scales used in the Norwegian CAMHS. RS-DBD measures inattention, hyperactivity, and impulsivity on 18 items with the same response options: (1) Never/Rarely, (2) Sometimes, (3) Often, (4) Very often (Table S1) [12]. We pooled data on ADHD symptoms for 2011–2016; birth years 2003–2008 (*n* = 39,850). 323 individuals in MoBa were dropped as they did not have data on municipality.

MoBa is a population-based pregnancy cohort study conducted by the Norwegian Institute of Public Health. Participants were recruited from all over Norway from 1999 to 2008. Women consented to participation in 41% of the pregnancies. The cohort includes 114,500 children, 95,200 mothers and 75,200 fathers [16]. MoBa was established with a license from the Norwegian Data Protection Agency and approval from The Regional Committees for Medical and Health Research Ethics (REK), and is now regulated by the Norwegian Health Registry Act. We use version 12 of the quality-assured data files released for research in January 2019, where geo-linkage was available for cohorts from 2002. This study was approved by REK (2017/2205).

### ADHD diagnosis

We used municipality-level data on all new patients registered with ADHD diagnosis in NPR (ICD-10, F90.0) between 2011 and 2016. We calculated the cumulative incidence proportion of ADHD among individuals aged 0–18 years, defined as the number of new ADHD diagnoses (*n* = 19,342) divided by the number of all individuals in that population using population data from Statistics Norway [17, 18]. For that purpose, we used the population mid-value for 2011–2016, conventionally defined for even numbers as the mean of the two mid-values (*n* = 1,189,496).

### Clinics’ catchment areas

Clinics are our unit of analysis because decision-making on diagnosis and potential treatment cultures manifest at clinic level. Clinics’ catchment area was inferred in collaboration with NPR using data on patient contacts at clinics by patients’ residence municipality in 2009. CAMHS are organized with clinics serving one or more municipalities (and/or city districts in Norway’s four largest cities) which comprise the clinics’ catchment area. The clinic a municipality is served by was defined as the clinic with the highest number of patient contacts from that municipality. For example, if a clinic in northern Norway is registered with 25 contacts from patients residing in a municipality in western Norway, and 800 contacts from patients residing in a municipality in northern Norway, the latter was defined as the main municipality the clinic serves. There were no major changes in municipality codes during the period of this study. Cities are represented by one clinic as we only have municipality-level data, reducing number of clinics from 73 to 63. The clinics catchment area list was quality-assessed by examining clinics’ own descriptions of catchment areas. When combining data from MoBa and NPR, six municipalities were not merged as these were not represented in MoBa in 2011–2016, giving a total of 416 municipalities. We use geographical data on latitude and longitude collected by Fiva et al. [19] to map and examine clusters of ADHD diagnosis. NPR data follow municipality classification per 2018 (*n* = 422) while the map data follow the municipality classification prior to 2018 (*n* = 428). For the map data, we adjusted for five municipality mergers providing six additional municipalities (*n* = 428) given same value as the municipality they were merged to. NPR is a health registry with information on all individuals who have received or are awaiting treatment in specialist healthcare services since 2008 [20]. (Figure 1A) is based on prevalence data on studies from the UK [21], Sweden [22], Finland [23], Greece [24], Ireland [25], and Norway [11], with the remaining countries covered in a comparative study [26].

### Statistical analyses

Confirmatory factor analysis (CFA) was applied to measure the latent ADHD symptoms construct from the symptoms score items in RS-DBD [27]. Goodness-of-fit statistics for the CFA used to measure ADHD symptoms aligns with commonly accepted values (CFI = 0.93, RMSEA = 0.07, SRMR = 0.05, *p* > *χ*^2^ =  < 0.0001; full model in Supplement). Data on symptoms and diagnosis of ADHD on the individual- and municipality-levels were aggregated to the clinic-level to examine between-clinics variation and associations between ADHD symptoms levels and ADHD diagnosis.

We examined the extent to which ADHD diagnosis and ADHD symptoms varied at clinic level by comparing observed proportions to expected values under ${\text{H}}_{0}$ of equal probability of diagnosis/symptoms across clinics. Confidence intervals under ${\text{H}}_{0}$ were bootstrapped using 10,000 draws from the binomial distribution with probabilities equal the grand mean. Observed proportions outside of the bootstrapped 95% CI were considered larger than chance variation.

Variance-components models were used to partition the variance in ADHD symptoms and ADHD diagnosis with municipality-level data nested within clinics. We examined variation in ADHD diagnosis and symptoms levels using the coefficient of variation (CV), a variability measure for the extent of variation relative to the mean calculated as the variable’s standard deviation (SD) divided by its mean value. Bootstrapping was used to derive the expected distribution of CV under ${\text{H}}_{0}$ of equal probability of symptoms/diagnosis across clinics. The observed CV was compared to the null distribution to examine the probability of observing the CV by chance under ${\text{H}}_{0}$.

We used fractional response regression models (FRM) [28] to test whether ADHD diagnosis is associated with ADHD symptoms. The mean incidence proportion of ADHD diagnosis was predicted by ADHD symptom levels in two separate models: one with 95th percentile cut-off and one with 90th percentile cut-off as the predictor. Heteroskedasticity-consistent standard errors were used. The model was weighted by number of MoBa-respondents within catchment areas. Average marginal effects (AME) were reported.

We examined the extent of unexplained variation in incidence of ADHD diagnosis by CV for the residual and for the squared correlation coefficient between the observed and the predicted values. To formally test whether the unexplained variation in ADHD diagnosis conditional on ADHD symptoms was larger than expected by chance, we compared the observed CV to the distribution of expected CVs under ${\text{H}}_{0}$, where ${\text{H}}_{0}$ was given by the predicted values from the FRM model. Since this prediction also contains statistical uncertainty, we conducted this analysis using a bootstrap approach.

## Results

The cumulative incidence of ADHD diagnosis was 0.016 (SD: 0.007, min–max: 0.004–0.039, IQR: 0.01–0.02) in 2011–2016. The proportion of children scoring over the 95th percentile on ADHD symptoms was 0.05 (SD: 01, min–max: 0–0.14, IQR: 0.045–0.053). For children scoring over the 90th percentile, the proportion was 0.1 (SD: 0.14, min–max: 0–0.14, IQR: 0.09–0.11). Two clinics had no MoBa-respondents scoring ≥ 95%, while one clinic had no participants scoring over ≥ 90%. There was nearly a tenfold difference in the incidence of ADHD diagnosis proportion from the clinic with the lowest to the highest level. (Figure 1) presents municipality-level geographical variation in the incidence rate of ADHD diagnosis showing clustering of areas with higher and lower levels of incidence of ADHD diagnosis. The intra-class correlation (ICC) from variance-components models for the incidence of ADHD diagnosis was 50.2% [CI 95%: 39 to 61] indicating that half of the total variance was attributed to the clinic level. The ICC for ADHD symptoms is < 0.01% for proportions of children with symptom scores ≥ 95% and 0.15% [CI 95%: 0.03 to 0.9] for proportions of children with symptom scores ≥ 90%.

**Fig. 2:**
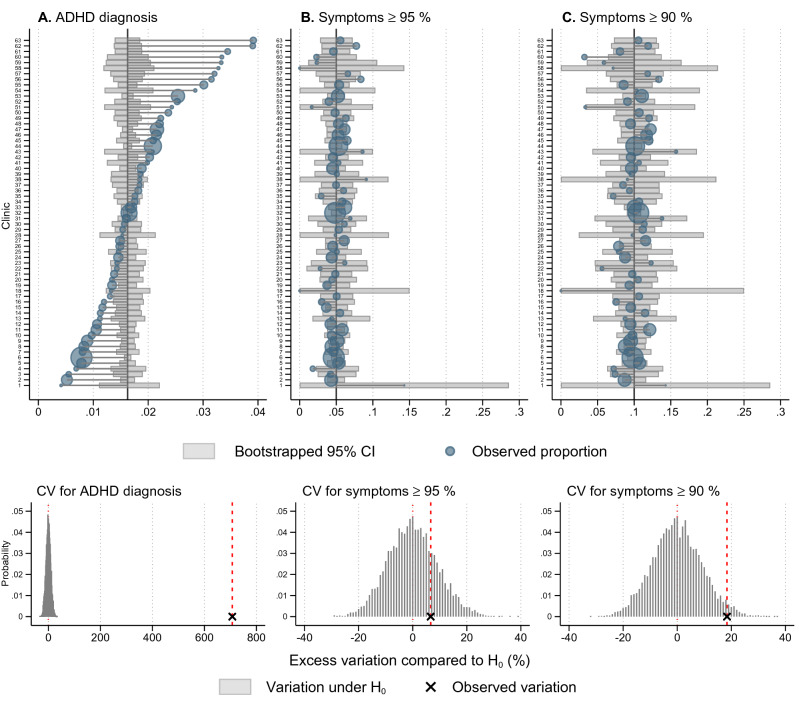
ADHD diagnosis incidence rate, ADHD symptoms ≥ 95% and ≥ 90% by clinics (*n* = 63), 2011–2016. Upper graphs in Panel (**A**–**C)** present bootstrapped 95% confidence intervals (CI) for chance variation around the population-weighted grand mean (black vertical line) for diagnosis, and sample-weighted grand mean for symptoms. Observed proportions (blue circles) outside 95% CI are larger than expected by chance. The lower graphs in Panel (**A**–**C**) present the observed coefficient of variation (CV) and the expected values of CV, under the null hypothesis that *the CV does not exceed chance variation*, based on 10,000 draws. The *x*-axis is the excess variation in CV compared to E(CV | H_0_)

In (Fig. 2), the upper graph in Panel A presents ADHD diagnosis proportions by clinics. The vertical line is the grand mean of observed population-weighted ADHD diagnosis with 95% CI for chance variation from 10,000 draws, whereas observed proportions (blue circles) outside 95% CI were larger than expected by chance. The observed coefficient of variation (CV) for the incidence of ADHD diagnosis proportions across clinics was 45.6% (*p* = 0).

The lower graph of Panel A presents the null distribution for CV with the excess variation in ADHD diagnosis compared to the mean of the null distribution measured in percentage difference. Similarly, the upper graph in Panel B presents proportions of children scoring ≥ 95th percentile for ADHD symptoms. Here, few observed proportions were outside 95% CI. The CV was 18% (*p* = 0.23), thus there was not support for more than chance variation as the observed CV was well within the null distribution (lower graph Panel B). In Panel C, there are more observed proportions of children scoring ≥ 90th percentile for ADHD symptoms compared to Panel B. The CV was 15% (*p* = 0.025), and there is evidence for more than chance variation.

From fractional response regression models (FRM) at the clinic level with the incidence of ADHD diagnosis as the outcome, the average marginal effect (AME) shows that the proportion of ADHD diagnosis increases 0.26 percentage points (95% CI: [0.09 to 0.42], *p* = 0.002) when the proportion of children and adolescents with ADHD symptoms ≥ 95% increase with one percentage point. We did not find support for an association between ADHD symptoms ≥ 90% and ADHD diagnosis (AME: 0.09, 95% CI: [− 0.06 to 0.24], *p* = 0.25) (Supplementary, Table S3).

**Fig. 3:**
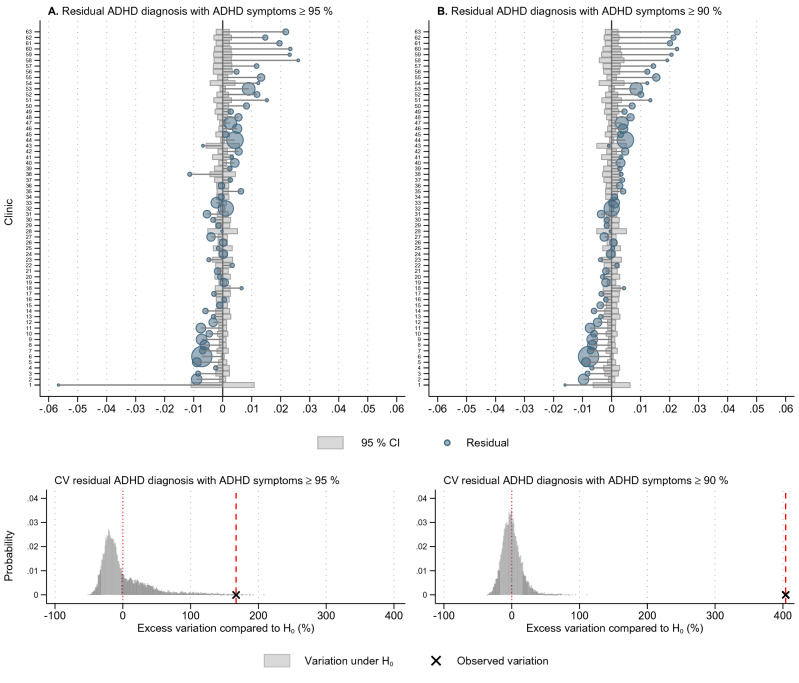
Differences in observed and predicted incidence rate of ADHD diagnosis by clinics (*n* = 63), 2011–2016. Upper graphs in panel **A** and **B** show residuals for the incidence of ADHD diagnosis after controlling for ADHD symptoms ≥ 95 and ≥ 90%, respectively. Clinics are sorted in ascending order by the incidence of ADHD diagnosis, with circles proportional to the population in catchment areas. 95% CI for residual centred at 0 for no difference between observed and predicted values. Observations outside 95% CI present differences not explained by ADHD symptoms in clinics’ catchment area. Lower graphs of panel **A** and **B** shows how much unexplained variation remains in ADHD diagnosis after controlling for ADHD symptoms. The extent of residual variation after controlling for ADHD symptoms is presented as a percentage difference from the expected value of CV, under the null hypothesis of *no remaining unexplained variation*, and is based on 10,000 draws

Predicted values from FRMs were used for analyses of unexplained variation (Fig. 3). The 95% CI were centered at 0 for no differences between observed and predicted values. There was large between-clinics variation in residuals, with few observed residuals in the 95% CI for chance variation for both models with proportions of ADHD symptoms ≥ 95% (Fig. 3A, upper graph) and ≥ 90% (Fig. 3B, upper graph) as predictors. Moreover, the observed CV for the residuals was considerably higher than the distribution of CVs under the null distribution for both models (Fig. 3A, B, lower graphs), which was supported by formal tests (Table 1). Overall, the residuals were still large after adjusting for ADHD symptoms ≥ 95% (or ADHD symptoms ≥ 90%), indicating that other factors are influential in explaining the remaining difference between observed and predicted proportions of ADHD diagnoses.

**Table 1:** Between-clinics variation in incidence rate for ADHD diagnosis, high levels of ADHD symptoms, and unexplained variation in ADHD diagnosis after controlling for ADHD symptom levels

Model	(1)	(2)	(3)	(4)	(5)
	ADHD diagnosis, unconditional	ADHD symptoms ≥ 95%, unconditional	ADHD symptoms ≥ 90%, unconditional	Residuals:ADHD diagnosis, conditional on symptoms ≥ 95%	Residuals:ADHD diagnosis, conditional on symptoms ≥ 90%
Coefficient of variation (CV)					
Observed CV	.46	.18	.14	.45	.44
Mean CV under ${\text{H}}_{0}$	.06	.17	.12	.17	.09
[Min, Max]	[.04–.08]	[.12–.24]	[.08–.16]	[.08–.54]	[.05–.18]
Test statistic: Percent deviation between observed CV and mean CV under H_0_	713.9	7.6	19.4	192.3	414.0
* p*-value	0	.23	.06	0	0
* R*^*2*^	–	–	–	.13	.04

The coefficient of variation (CV) shows how much variation there is relative to the mean and is calculated as the variable’s standard deviation divided by its mean value. ${\text{H}}_{0}$ for CV is that variation does not exceed chance variation. *p* value is proportion of expected values under ${\text{H}}_{0}$ with values equal to, or above, observed value from 10,000 trials. Models 4 and 5 are weighted by participants in MoBa. *R*^2^ from fractional regression models with diagnosis as response and symptom levels as explanatory variable

## Discussion

### Summary of findings

We found support for large between-clinics variation in the incidence rate of ADHD diagnosis and considerably less variation in high levels of ADHD symptoms at the ≥ 90 percent level. There was no evidence for more than chance variation in symptoms at the ≥ 95 percent level. Municipalities clustered into areas with higher and lower levels of ADHD diagnostic incidence, where half of this variance could be ascribed to the clinic level. While there was evidence for a positive association between the incidence rate of ADHD diagnosis and high levels of ADHD symptoms, the explained variance in the incidence rate of ADHD diagnosis after controlling for ADHD symptoms was low.

### Strengths and limitations

There are two considerable strengths to this study. First, the analyses are based on a unique combination of nationwide geo-coded data on both symptoms and diagnosis of ADHD. We are not aware of other similar data sources that can be used to examine the research questions in this study, nor have we discovered any study on within-country variation in ADHD diagnosis that includes data on ADHD symptoms. Second, the Norwegian context is ideal due to the single provider healthcare system with only a small portion of patients using private sector healthcare reducing concerns of selection biases into healthcare.

There are limitations to consider. First, ecological bias may be a concern as both symptoms and diagnosis of ADHD are individual-level data aggregated to clinic-level variables. However, we examined clinic-level variation and associations and did not draw inferences for the individual level [29]. Second, statistical bias could be introduced by the modifiable areal unit problem since several units of observation can be used [29]. Clinics are arguably more relevant compared to other area definitions as patients are diagnosed at clinics and there may be local treatment cultures [30]. Third, the association between symptoms and diagnosis of ADHD may be subject to confounding bias either toward or away from the null. The proportion of individuals with high levels of ADHD symptoms includes treated and untreated ADHD, where the former reduces symptoms and the association between symptoms and diagnosis. As well, population composition and other potential confounders may vary between clinics. Moreover, ADHD is highly heritable (88%) [31]. Siblings live in the same catchment area which may inflate familial risk factors for ADHD. However, the focus of this study is the unconditional, and conditional on ADHD symptoms, between-clinics variation in ADHD diagnosis. Fourth, areas with high levels of ADHD diagnosis may raise awareness and increase parent- and teacher reporting of ADHD symptoms and referral rates to specialist health services, causing a reverse causal path between rates of diagnosis and ADHD symptoms. There is currently no strong empirical evidence supporting this concern. Fifth, there are at least two potential sources of selection bias. MoBa may be affected by sampling bias with overrepresentation of individuals with high SES [32], and underrepresentation of non-Norwegians, young females, single households, mothers with > 2 births or previous stillbirths, and smokers [33]. NPR only includes patients in the specialist health services and lower SES predicts more health services use [34]. Both selection mechanisms can affect observed variations and associations between symptoms and diagnosis of ADHD. Sixth, a concern may be chance findings, e.g., due to sample size, statistical power, or researcher degrees of freedom. While the Type 1 error rate is constant in increasing sample size, the Type II error rate decreases. Thus, if this study is underpowered, there is no way of knowing whether failing to reject the null hypothesis is due to insufficient sample size or a real lack of effect. As the sample consists of clinics in Norway, we could only increase the sample size using city–district codes for the four largest cities, which we did not have access to. Sixth, our measure of ADHD symptoms is only restricted to children when they are 8 years old. While this corresponds with the mean age at diagnosis, it may not perfectly reflect symptom levels for the children and adolescents from 0 to 18 years whom we have diagnosis data on.

### Contribution and interpretation

This is the first study to combine nationwide data on both symptoms and diagnosis of ADHD to examine the extent to which within-country variation in ADHD diagnosis is explained by ADHD symptoms. We find considerable between-clinics variation in ADHD diagnosis despite free access to healthcare, a comprehensive welfare state, and comparatively low social inequality, which reduces the potential impact of socioeconomic conditions. This finding is in line with existing research on within-country variation in ADHD diagnosis, where clusters of municipalities with high and low incidence of ADHD diagnosis have been identified [4–6]. Regional differences in diagnostic practice have been presented as the most plausible explanation in another Norwegian study on geographic variation in ADHD diagnosis [6]. A survey supports that clinician’s policy toward ADHD treatment varies [35]. The main question from a health policy perspective is whether the observed variation is unwarranted or fully explained by patient and provider characteristics [30]. The high remaining residual variation in ADHD diagnosis after controlling for ADHD symptoms suggests that other factors are important drivers of between-clinics variation in ADHD diagnosis.

### Implications

ADHD symptoms should arguably explain a considerable part of between-clinics variation in ADHD diagnosis since the diagnosis is based on the assessment of symptoms, functional impairment, and differential diagnosis. The inherent puzzle clinicians are faced with in diagnosing patients with symptoms around the threshold for diagnosis may introduce a random component in being diagnosed with ADHD based on the patient’s geographical residence. Accordingly, for some patients, being diagnosed with ADHD and receiving ADHD medication may ultimately come down to residing in one catchment area rather than another. From a health policy perspective, this is worrisome as it challenges the principle of equal healthcare regardless of geography. From a research perspective, the between-clinics variation in ADHD diagnosis presents a potential quasi-experiment that can inform clinical practice on effects of ADHD diagnosis and treatment [5, 36]. Future research may consider a quasi-experimental approach that exploits geographical variation in diagnosis or medication rates to fill knowledge gaps that are challenging to address with randomized experiments.

## Supplementary Information

Below is the link to the electronic supplementary material.

Supplementary file1 (PDF 295 KB)

## Data Availability

Data cannot be publicly shared due to strict data privacy laws. Data from the Norwegian mother, father and child cohort study (MoBa) and the Norwegian Patient Registry can be made available by application to the data owners.
