# Supplementary material for: Geographical variation in ADHD: do diagnoses reflect symptom levels?
Source: Eur Child Adolesc Psychiatry. 2022 May 18;32(9):1795–803. doi: 10.1007/s00787-022-01996-7 (PMC10460326; doi:10.1007/s00787-022-01996-7)
Supplement: Supplementary file 1 — Supplementary file1 (PDF 295 KB) [file 787_2022_1996_MOESM1_ESM.pdf]

Supplementary

1. Data used in confirmatory factor analysis for ADHD symptoms

Variable	Item		Factor
NN119	1	Fails to give close attention to details or makes careless mistakes in schoolwork	Attention-deficit
NN120	2	Has difficulty sustaining attention in tasks or play activities	
NN121	3	Does not seem to listen when spoken to directly	
NN122	4	Does not follow through on instructions and fails to finish school work, chores or duties (not due to oppositional behaviour or failure to understand instructions)	
NN123	5	Has difficulty organizing tasks and activities	
NN124	6	Avoids, dislikes or is reluctant to engage in tasks that require sustained mental effort (such as schoolwork or homework)	
NN125	7	Loses things necessary for tasks or activities (pencils, books, toys)	
NN126	8	Is easily distracted	
NN127	9	Is forgetful in daily activities	
NN128	10	Fidgets with hands or feet or squirms in seat (sits uneasily)	Hyperactivity/ impulsivity
NN129	11	Leaves seat in classroom or in other situations in which remaining seated is expected (e.g. at the table or in group gathering)	
NN130	12	Runs about or climbs excessively in situations in which it is inappropriate	
NN131	13	Has difficulty playing or engaging in leisure activities quietly	
NN132	14	Is "on the go" or acts as if "driven by a motor"	
NN133	15	Talks excessively	
NN134	16	Blurts out answers before questions have been completed	
NN135	17	Has difficulty awaiting turn	
NN136	18	Interrupts or intrudes on others, such as in conversation or play	

Table S1. MoBa data on ADHD symptoms. Response options (all items): 1 = Never/rarely; 2 = Sometimes; 3 = Often; 4 = Very often.

2. Additional information on statistical analyses

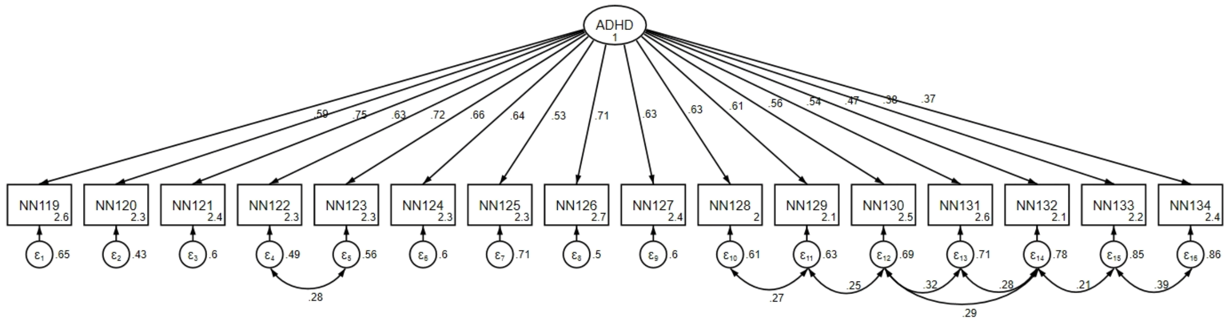

Figure S1. Confirmatory factor analysis. Standardized. Based on NN119-NN136 for years 2011-2016 (n = 39,850).

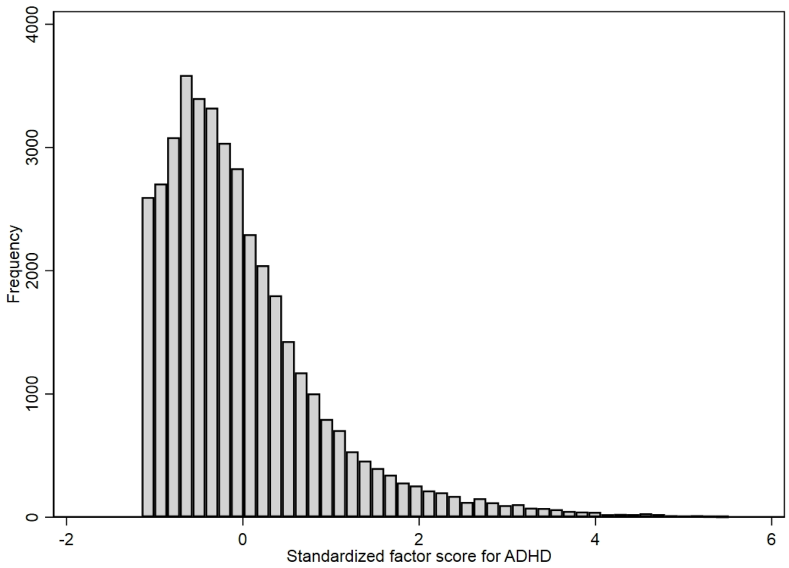

Figure S2. Histogram of factor score for ADHD symptoms.

**Fit statistic**	**Value**
Likelihood ratio	
χ^2 ms, model vs. saturated	18620.4
$p > \chi^2$	< 0.001
χ^2 ms, baseline vs. saturated	246434.6
$p > \chi^2$	< 0.001
RMSEA	0.07
CFI	.93
SRMR	.05

Table S2. Goodness of fit for confirmatory factor analysis of ADHD symptoms.

Model<br>Incidence of<br>ADHD diagnosis	(1)<br>Symptoms<br>≥ 90 %	(2)<br>Symptoms<br>≥ 95 %	(3)<br>Symptoms<br>≥ 90 %	(4)<br>Symptoms<br>≥ 95 %
AME	.01	.06	.09	.26
95% CI	[-.08, .1]	[-.09, .21]	[-.06, .24]	[.09, .42]
Delta SE	.04	.08	.08	.08
Z	.23	.74	1.15	3.05
P > | z |	.82	.46	.25	.002
Weights	No	No	Yes	Yes

Table S3. Average marginal effects from fractional response models. Incidence of ADHD diagnosis regressed on proportion with high levels of ADHD symptoms at clinic level. Models are weighted by number of participants in MoBa in clinics' catchment area. Abbreviations: AME = average marginal effect, Delta SE = delta method standard error, CI = confidence interval.
